# A Multidisciplinary Approach and Development of an Algorithm for Timely Repair of Central Venous Access in Pediatric Patients

**DOI:** 10.7759/cureus.23355

**Published:** 2022-03-21

**Authors:** Kasia Wallace-Shaw, Ayoola Adigun, Anisha Mohandas, Amanda Costa, Michele Markley, Debora Duro

**Affiliations:** 1 Pediatrics, Broward Health Salah Foundation Children's Hospital, Fort Lauderdale, USA; 2 Pediatric Surgery, Broward Health Salah Foundation Children's Hospital, Fort Lauderdale, USA; 3 Pediatric Gastroenterology, Broward Health Salah Foundation Children's Hospital, Fort Lauderdale, USA

**Keywords:** protocol, intestinal failure, treatment algorithm, catheter complication, central-line associated bloodstream infection

## Abstract

Background: Central venous catheters (CVCs) carry a risk for many complications. This can lead to numerous and prolonged hospitalizations for patients undergoing intravenous nutrition. The aim was to create a standardized protocol for the medical facility to expedite the repair process as well as implement a broadened educational effort for the care of CVCs.

Method: A retrospective chart review was completed for 365 catheter days before implementation. Two protocols were then created in collaboration with the multidisciplinary team. Prospective chart data were subsequently collected 365 catheter days post-implementation.

Result: Pre-implementation (32 encounters), 100% of compromised CVCs required admission. Post-implementation (21 encounters), only 48% of compromised CVCs required admission accompanied by an overall reduction in the number of compromised catheters that presented to the hospital. The average hospital length of stay pre-protocol initiation decreased from 7.2 to 1.8 days post-protocol initiation. The implementation of our algorithm also lead to a decrease in the average cost of compromised CVC repair inpatient ($2741) vs repair in the emergency department ($34,436).

Conclusion: This study demonstrates that working with a multidisciplinary team utilizing a standardized protocol improved the quality of patient care by decreasing hospital admissions for compromised CVCs. The authors also conclude that the expedited repair of CVCs can help alleviate health care costs for both families and the hospital system.

## Introduction

Pediatric intestinal failure patients undergoing multidisciplinary rehabilitation are dependent on central venous catheters (CVCs) for intravenous nutrition and medication administration. Pediatric intestinal failure (IF) is characterized by the inability of the gastrointestinal tract to absorb adequate nutrition and fluid to maintain hydration and support growth resulting in the need for long-term parenteral nutrition (PN) delivered via a CVC [1]. Long-term CVC usage can result in various complications, including central-line associated bloodstream infection (CLABSI), venous thrombosis, and catheter compromise [2,3]. Catheter compromise can include urinary tract infection, obstruction, urinary leakage, and genitourinary tract trauma [4]. These complications over time can result in loss of access due to vessel depletion resulting in patients being unable to receive necessary intravenous nutrition [5,6]. A study was carried out involving central line catheters which reported that central line breakage accounted for 12% of all catheter-related complications in their sample population, with smaller lumen catheters (6.6Fr) being less successfully repaired than larger lumen catheters (9.6Fr) [7]. All patients in our study had the same catheter type and size because a standard lumen catheter size is used at our healthcare institution.

The practice gap that exists outside of the authors’ hospital is the lack of standardized protocol and guidelines for CVC repair. During extensive literature review using multiple databases like PubMed® and Google Scholar, the researchers did not find any original published research manuscript that describes a standardized algorithm to expedite CVC repair. The United States Centers for Disease Control and Prevention (CDC) recently reported nearly 60% reduction in CLABSIs, which shows the results of a national initiative and focus on improving the overall patient safety, public reporting of infection rates, and a national campaign supported by the Agency for Healthcare Research and Quality (AHRQ) [8]. During several attempts to prevent and reduce intravascular catheter-related infections, the CDC has developed specific guidelines which help to synthesize current evidence on the delivery of care to patients with CVC due to their widely acknowledged effective role in control and reduction of catheter-related infections [9,10]. There have been several studies done by others demonstrating that the successful implementation of guidelines for overall best practices when combined with effective strategies aimed at improving management will result in the reduction and improvement of infection rates [11,12].

Based on the authors’ experience at Florida Intestinal Rehabilitation, Support and Treatment Program (FIRST), nonfunctional CVCs often require inpatient admission for repair and alternative intravenous access for nutrition. Furthermore, nonfunctional CVCs which are a result of device dysfunction can produce complications that include fibrin sheath formation, fracture, thrombosis, central venous stenosis, and infection [13]. Catheter fracture which can occur in subclavian lines after a catheter has been in place for an extended period can lead to rare but potentially fatal serious complications associated with high morbidity and mortality related to catheter embolization including sepsis, endocarditis, cardiac perforation, or arrhythmia [13,14]. Furthermore, every prior line break required inpatient admission for the surgeon to repair at the medical center. The authors are aware that at other medical centers, this is not the case which is why they wanted to spearhead this change. This can result in a significant financial burden for intestinal rehabilitation patients and families, resulting in billions of dollars of health care costs annually [15,16]. In many instances, obtaining peripheral access in these specific patients can be extremely difficult, since it has been found that venous compromise is prevalent in patients who require chronic access with approximately 50% of intestinal failure and renal failure patients having obstruction of at least one major venous pathway [17]. In addition, in the 30 days following a CVC repair, an earlier study showed that patients are at a greater risk for bacteremia and CLABSI than they would have otherwise been exposed to with routine care of the line [18,19]. While more recent studies have assessed CLABSI rates after CVC repair and shown no increase in the risk of infection after the procedure [20,21]. This study reports the development of a standardized protocol at Florida Intestinal Rehabilitation Support and Treatment Program (FIRST) at the Salah Foundation Children’s Hospital for the expedited repair of compromised CVC in pediatric intestinal rehabilitation patients.

## Materials and methods

The authors conducted a retrospective, prospective chart review from the electronic health records and included demographics, height, weight, primary diagnosis, hospitalization for CVC repair, and length of stay during hospitalization. The study's protocol was expeditiously approved by the institutional review board (IRB) and ethics committee associated with Broward Health (approval number: 2019-078). IRB approval was obtained from Broward Health Medical Center for 11 patients undergoing treatment at FIRST for one year (365 catheter days) before the start of the study.

Before this project, every patient who had a CVC break which includes peripherally inserted central catheter (PICC) lines and other central venous lines (CVLs) at the healthcare institution was hospitalized. A total of 365 catheter days (July 2019 retrospective data) was chosen because this is the timeframe preferred at the hospital since patients had catheters long before and after the study. Prospective electronic health record (EHR) chart review was started after the commencement of two evidence-based algorithms for the repair of compromised catheters, as well as increased educational strategies for the proper care of those catheters that were already compromised. The decision processes or strategies employed to facilitate the steps in the algorithm included the stepwise procedure for ER repair, and admission for repair. Depending on the case and presenting factors, the authors picked which algorithm is appropriate and followed it through. The researchers searched for existing protocols for repair of CVC and did not find any published original research manuscript describing this procedure.

**Figure 1:**
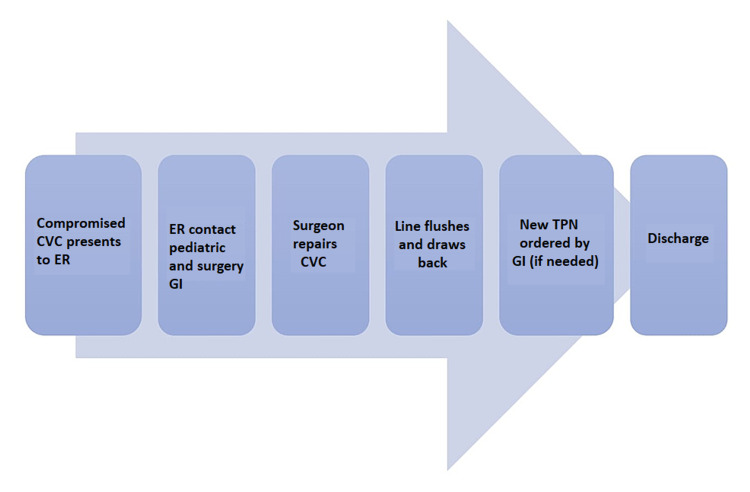
Delineating CVC immediate repair in the ER CVC: central venous catheter; ER: emergency room; GI: gastrointestinal; TPN: total parenteral nutrition

Monthly seminars were held with the nursing staff at the healthcare facility to provide education on the care and management of CVCs. A proportion of patients with intestinal failure have an external CVC which requires routine maintenance. Thus, caregivers (which includes parents and home health care nurses) are expected to learn how to care for CVCs at home, or in a home-like environment. Training sessions were implemented with the inpatient nursing team as well as extra educational sessions with parents and home nurses in the clinic setting. Caregivers receive the required education during their first inpatient admission and are expected to achieve skill mastery to prevent CVC complications, including CLABSIs. At our hospital institution, the triage for discharge planning includes ensuring parents attend at least one educational session on CVC care. Parents’ CVC care education is conducted in a formal class setting by a nurse with strong organizational and leadership skills, or with the patient by the bedside, where the caregiver demonstrates how CVCs are taken care of. Figure 1 shows how CVCs undergo immediate repair in the ER.

**Figure 2:**
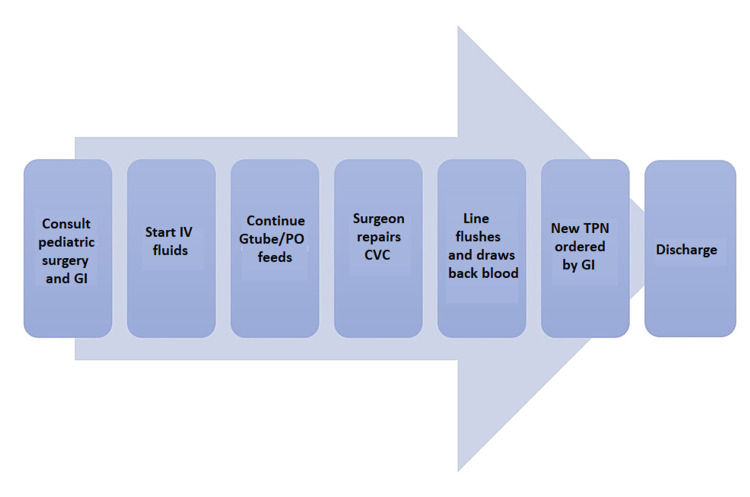
CVC repair requiring inpatient admission CVC: central venous catheter; GI: gastrointestinal; IV: intravenous; Gtube: gastrointestinal tube; PO: per os; TPN: total parenteral nutrition

Figure 2 shows how the processes for catheter repair that requires hospitalization and all the way to discharge. Algorithm 2 was used to ensure CVCs were repaired within 24 hours when patients required inpatient admission to the pediatric unit (Figure 2).

**Figure 3:**
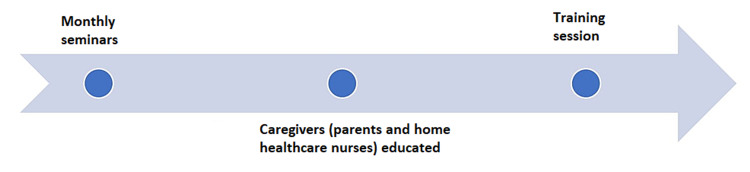
Timeline showing educational interventions during study implementation

Figure 3 shows the timeline (July 2019 to June 2020) of educational interventions done during study implementation. This was done to further illustrate our addition to the implementation of the work process algorithm which will be beneficial. It also helps to show how we have drastically decreased secondary compromises as a result of the educational intervention. 

There are several factors that could potentially contribute to the failure of successfully implementing algorithm 1 and they include presentation at odd hours, unavailability of surgical team, and unsuccessful repair of catheter at the ER. An unsuccessful ER repair is defined as when a line does not flush or draw back blood. The multiple attempts at repair involve the first repair leaking after flushing attempts and the surgeon opens a new kit to attempt repair again. Two to three attempts were typically made before the decision to admit the patient to the pediatric unit for further care. With each algorithm, it is required that the pediatric gastroenterologist is made aware of the repair to order total parenteral nutrition (TPN) for the patient upon discharge. New TPN rates may be required for that evening until the repair is fully set. After the CVC is repaired, it is not used until after six hours at which it is run at half rate as per the surgeons' recommendation. After 12 hours, it is then run at full rate.

## Results

A total of 11 patients were enrolled in July 2019 for 365 catheter days of data collection. Patient demographics include male (27%), female (73%), with a mean weight and height of 14.1 kg and 91.2 cm, respectively, as shown in Table 1. 

**Table 1:** Characteristics of patients enrolled In July 2019 for 365 catheter days N: number

Demographics	Number (N)	Percentage (%)
Male	3	27
Female	8	73
-	Average	Z-score
Weight (kg)	14.1	-1.31
Height (cm)	91.2	-0.96

The age of the patients ranged from four months to 16 years. Primary diagnoses include necrotizing enterocolitis, intestinal atresia, Hirschsprung’s disease, gastroschisis, and intestinal pseudo-obstruction. Before initiation of the protocol, 100% of the 32 encounters for CVC compromise required inpatient admission (Table 2). Post-initiation of interventions, only 48% (July 2019 to June 2020) of encounters required hospitalization (Table 2).

**Table 2:** Results of the study comparing the pre- and post-protocol initiation N: number

Variables	Pre-protocol initiation	Post-protocol initiation
Admission rate (%)	100	48
Number of ED repairs (N)	1	9
Hospital length of stay (average)	7.2	1.8

There were a total of 21 encounters in the 365 catheter days post-initiation, with 11 encounters repaired using algorithm 1. The remaining 10 encounters resulted in eight inpatient admission, one outpatient surgery repair, and one repair at a sister hospital. One patient required outpatient surgery repair because the surgeon was available at the time of presentation to take the patient to the operating room (OR). One repair at a sister hospital post-initiation of the protocol was done because that was where the patient presented for their healthcare problem, i.e., compromised CVC. Every repair was completed by the Salah Foundation Children’s Hospital pediatric surgery team.

Retrospective data collection also indicated multiple instances of secondary compromise after primary repair. The secondary compromise includes distal occlusions, improper dressing application resulting in catheter breakage, and improper line care resulting in secondary breakage. After protocol initiation and the education of emergency department (ED) and inpatient nursing teams, there were no instances of secondary compromise. Manipulation of the central line also places patients at increased risk of CLABSI and in the 365 catheter days before the protocol, there were three reported CLABSI within seven days of catheter repair. Following post-protocol initiation, which is the period of 365 days, there have been zero reported CLABSI. The mean hospital length of stay (LOS) decreased from approximately seven to two days after the standardization of care. Patients needed to stay an average of 7.2 days before the standardization of care typically due to infection, secondary line break, other medical conditions being addressed, or coordination of care for discharge. Cost analysis was provided for each admission, with an average of $34,436. In comparison, repairs in the emergency department cost an average of $2741 per visit. The average costs of both admission and repair in the emergency department were provided by the finance department of the hospital. The Financial Identification Number (FIN) was sent to the finance department and the cost analysis was obtained from them.

## Discussion

To the best of our knowledge, this is the first study to examine the use of a standardized protocol and guideline for CVC repair. To provide high-quality, cost-effective care, an evidence-based protocol for the expedited repair of compromised CVCs at FIRST was developed. This retrospective, prospective cohort study indicates that the protocol is an effective guideline for the treatment of nonfunctional catheters. Retrospective chart review was indicative of prolonged hospitalizations, multiple instances of secondary catheter compromise, cost-ineffectiveness, and multiple instances of suspected iatrogenic CLABSI. The protocol alleviated the fiscal implications along with the adverse events to patients and their families by educating the medical staff on the proper management of central lines and shortening time to CVC repair. As a result, the authors have seen a significant decrease in admission rates, length of hospitalization, and infection rates.

The multidisciplinary team approach for pediatric intestinal rehabilitation has been proven to be a successful treatment approach for this patient population. At FIRST, the multidisciplinary team includes pediatric residents, nurses, general pediatricians, pediatric surgeons, pediatric gastroenterologists, and pediatric emergency physicians. A standardized protocol was devised by the aforementioned multidisciplinary team. This standardization allowed for streamlining patient care from their initial presentation to the ER. Creating a protocolized chain of events ensured that the surgeons and gastroenterologists were all contacted in a timely fashion, in an attempt to expedite the repair and the ordering of home parenteral nutrition. A series of educational workshops for central line care were provided for all pediatric nurses in the facility as well.

The data demonstrates a decreased length of stay, secondary compromise, reduction in the rate of catheters that have already been compromised. The decrease in LOS and secondary compromise can be attributed to increased educational efforts, ensuring all clinical staff provided appropriate line care. The overall decrease in compromised CVC further reflects the educational efforts of this study, as parents and home-health caregivers were routinely re-educated on CVC care at the time of discharge. The authors were also able to demonstrate a decrease in post-repair CLABSIs. The authors believe this is attributed to the education efforts both in the inpatient setting and the home setting to the patient’s caregivers on proper care and management of the CVC.

The extensive study of our data suggests that the use of evidence-based algorithms helps to not only reduce healthcare costs to the patients and their caregivers/families but also demonstrates a positive financial impact on the hospital, and health care system as a whole.

Study limitation

This study is limited by its retrospective, prospective design and was restricted to 365 days before and after protocol implementation, and also has a relatively small sample size. Therefore, the study was not designed to detect short-term outcomes or adverse sequelae in the days following the implementation of an algorithm for CVC repair. The findings from this study warrant further investigation into the development and implementation of an algorithm for the early repair of CVCs involving larger sample sizes so that the results could be reproduced across the various hospital systems in the country. In addition, although the study population represents it is single-centered, the authors’ hospital catchment area includes a diverse and large geographic segment of the United States and focuses on medically complex, chronically ill children, who are the most likely out of the pediatric population to be affected by CVC breakage and CLABSI, and thus the findings might not be generalizable to other settings or geographic location.

## Conclusions

The results of this study show that the expedited repair of CVCs in the ER leads to a reduction in the number of inpatient admissions and the average length of hospital stay. The implementation of CVC repair was shown to be a highly successful procedure with a low risk of infection. The development of an algorithm for CVC repair is crucial to improving the quality of life for children living with intestinal failure. In addition, the cost benefits of having timely CVC repair in the hospital cannot be overemphasized as this could lead to an overall increase in patient satisfaction and health outcomes.
